# A Case of Chronic Expanding Hematoma Following Extraperiosteal Paraffin Plombage After 50 Years

**DOI:** 10.7759/cureus.81444

**Published:** 2025-03-29

**Authors:** Daiki Hayashi, Naoko Ose, Hideki Nagata, Eiichi Morii, Yasushi Shintani

**Affiliations:** 1 Department of General Thoracic Surgery, Osaka University Graduate School of Medicine, Suita, JPN; 2 Department of Pathology, Osaka University Graduate School of Medicine, Suita, JPN

**Keywords:** chronic empyema, chronic expanding hematoma, complication, curettage, paraffin plombage, surgical excision

## Abstract

Chronic expanding hematoma (CEH) is a late complication of extraperiosteal paraffin plombage. Various late complications have been reported with this historical procedure, including expanding hematoma, paraffinoma, blood specimens complicating chronic empyema, and malignant tumors. A 75-year-old male presented with left-sided lateral chest pain. Fifty years prior, he had undergone a left upper lobectomy with extraperiosteal paraffin plombage for pulmonary tuberculosis. Three years before the current presentation, he had been admitted to another hospital with fever. At that time, a chest computed tomography (CT) scan showed the expanding plombage and a mass in the plombage. Upon referral to our institute, contrast-enhanced CT showed strong enhancement of part of the mass, and a CEH was suspected. The paraffin and hematoma were surgically removed. Pathological diagnosis was a CEH. Plombaged paraffin can lead to both malignant and benign complications even decades after the initial procedure. Surgical excision should be considered in symptomatic cases or when there is an expanding mass in the plombage space.

## Introduction

Extraperiosteal paraffin plombage was used as a collapse therapy for pulmonary tuberculosis before the development of anti-tuberculosis drugs, which were invented by Adams et al. in the 1950s [1]. However, late complications such as expanding plombage, paraffinoma, blood specimens complicating chronic empyema, and chronic expanding hematoma (CEH) have been reported [2-6]. We report a case of CEH developing 50 years after extraperiosteal paraffin plombage.

## Case presentation

A 75-year-old male had undergone left upper lobectomy and extraperiosteal paraffin plombage for treatment of tuberculosis 50 years ago. He was referred to our institute with left lateral chest pain when sneezing or coughing for a month. Laboratory investigations revealed a normal soluble interleukin (IL)-2 receptor level of 465 U/mL and an elevated neuron-specific enolase (NSE) level of 14.7 ng/mL, with no other abnormal findings.

**Figure 1:**
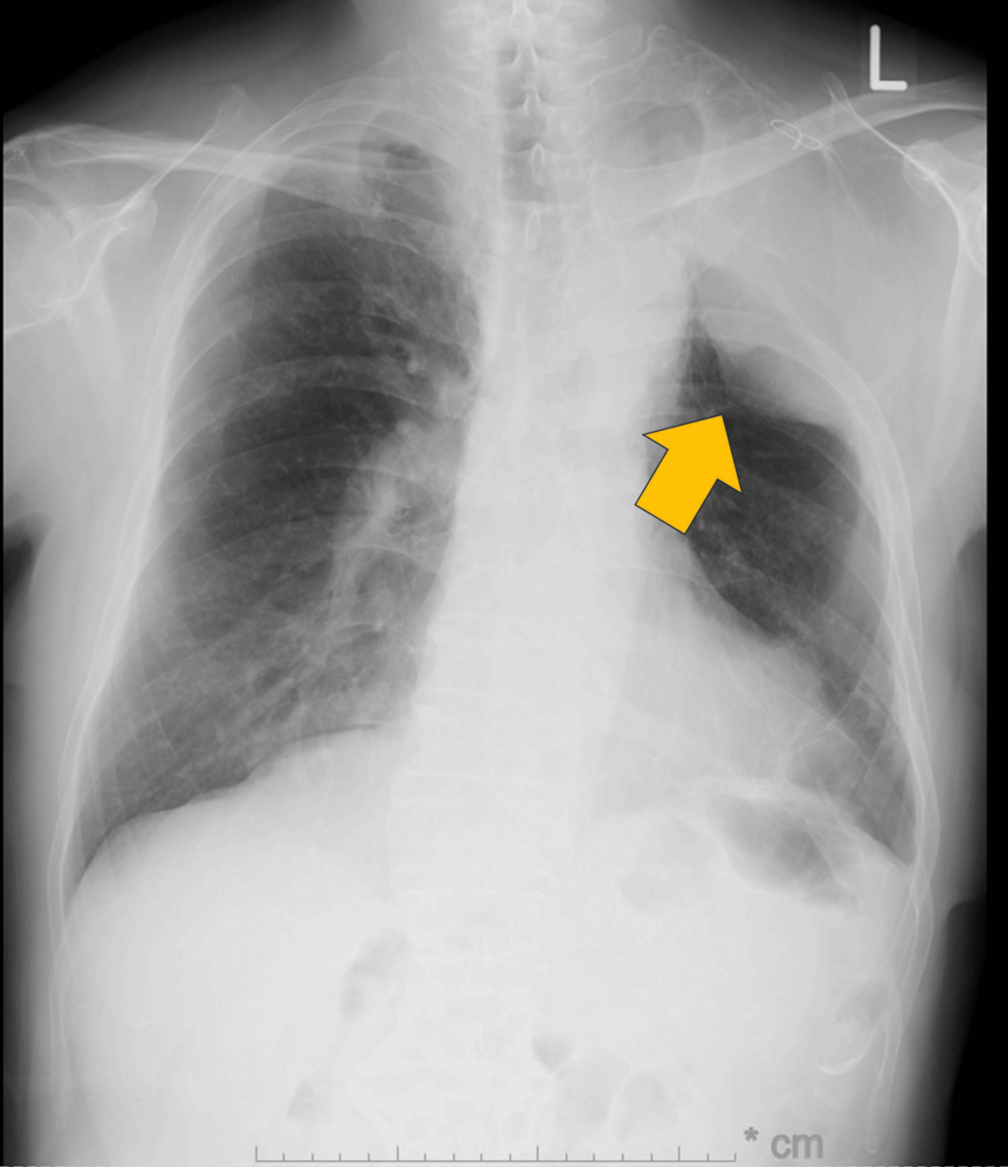
Chest X-ray findings Extraperiosteal paraffin plombage space was observed in the left upper lung field (arrow).

Chest X-ray showed an extraperiosteal paraffin plombage space in the left upper lung field (Figure 1).

**Figure 2:**
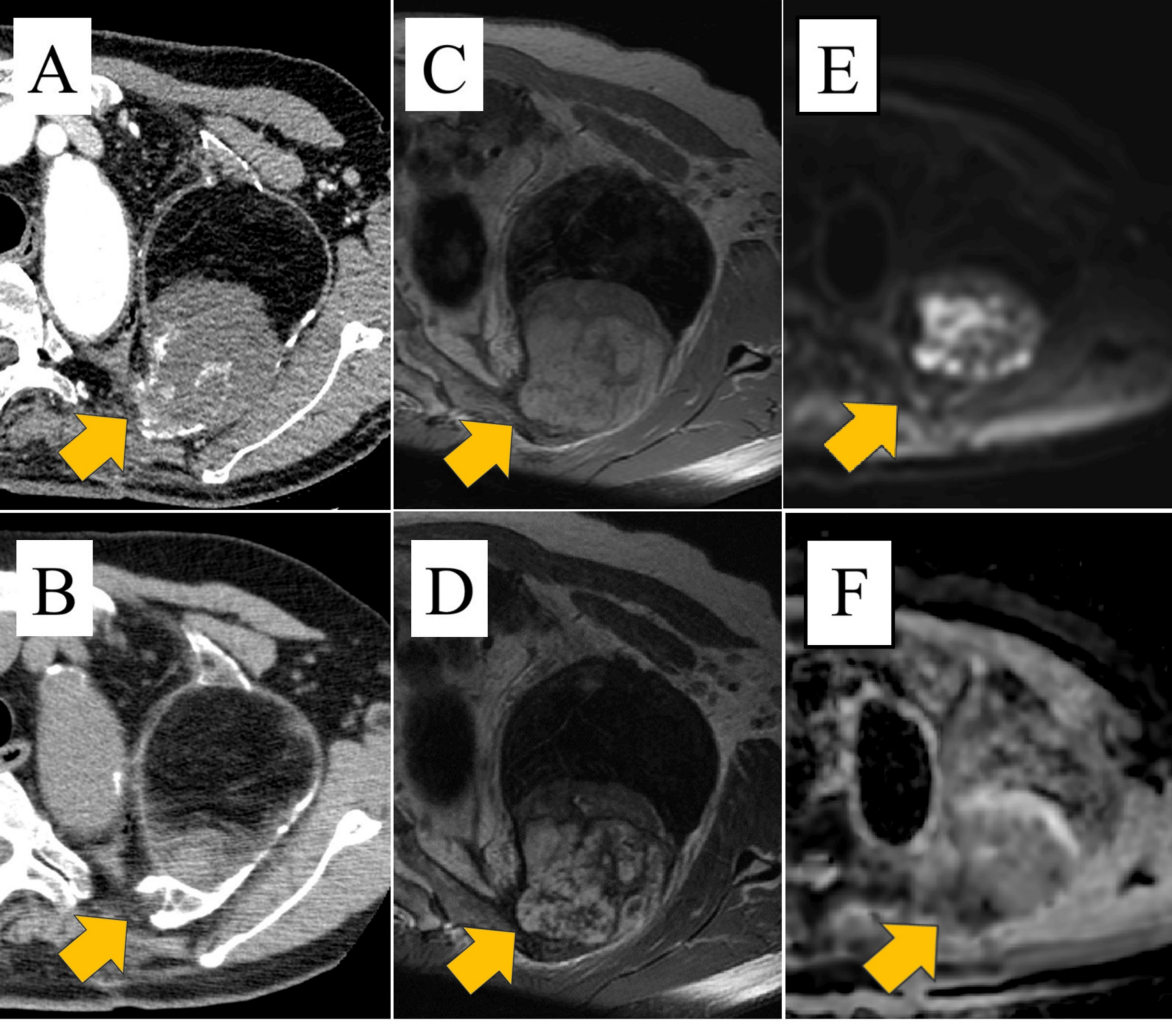
Chest CT and chest MR findings (A) Chest contrast-enhanced CT scan showing a plombage space with a 120 × 90 × 70 mm plombage space and a mass partly accompanied by a strong contrast effect in the plombage space (arrow). (B) A chest plain CT scan showed an 81 × 81 × 63 mm plombage space, and the size of the mass was 24 × 18 × 18 mm three years ago (arrow). (C) T1-weighted MR image showing a low-to-slightly high-intensity mass in the plombage space (arrow). (D) In the T2-weighted MR image, the mass had a low to high intensity and a mosaic-like appearance (arrow). (E) Diffusion-weighted imaging showed a mosaic appearance ranging from high to low signal intensity (arrow). (F) The apparent diffusion coefficient (ADC) map also showed a mosaic appearance ranging from high to low signal intensity (arrow).

There were no significant changes in X-ray over the previous year. Chest contrast-enhanced computed tomography (CT) scan showed a 120 × 90 × 70 mm plombage space with calcification at the margins on the left chest wall and within the space, and a 70 × 65 × 52 mm spherical mass in contact with fat and the chest wall (Figure 2A). The mass was partially strongly contrasted. The mass and plombage space had expanded compared with the plain chest CT scan performed three years earlier (Figure 2B). Magnetic resonance imaging (MRI) showed a mass with low-to-slightly high intensity on T1-weighted images (Figure 2C) and a mosaic-like appearance with low-to-high intensity on T2-weighted images (Figure 2D). Diffusion-weighted imaging showed a mosaic appearance ranging from high to low signal intensity (Figure 2E). The apparent diffusion coefficient (ADC) map also showed a mosaic appearance ranging from high to low signal intensity (Figure 2F).

Based on the enlarged plombage cavity, mass characteristics, and elevated NSE on blood tests, we suspected a CEH or pyothorax-associated lymphoma after paraffin plombage.

**Figure 3:**
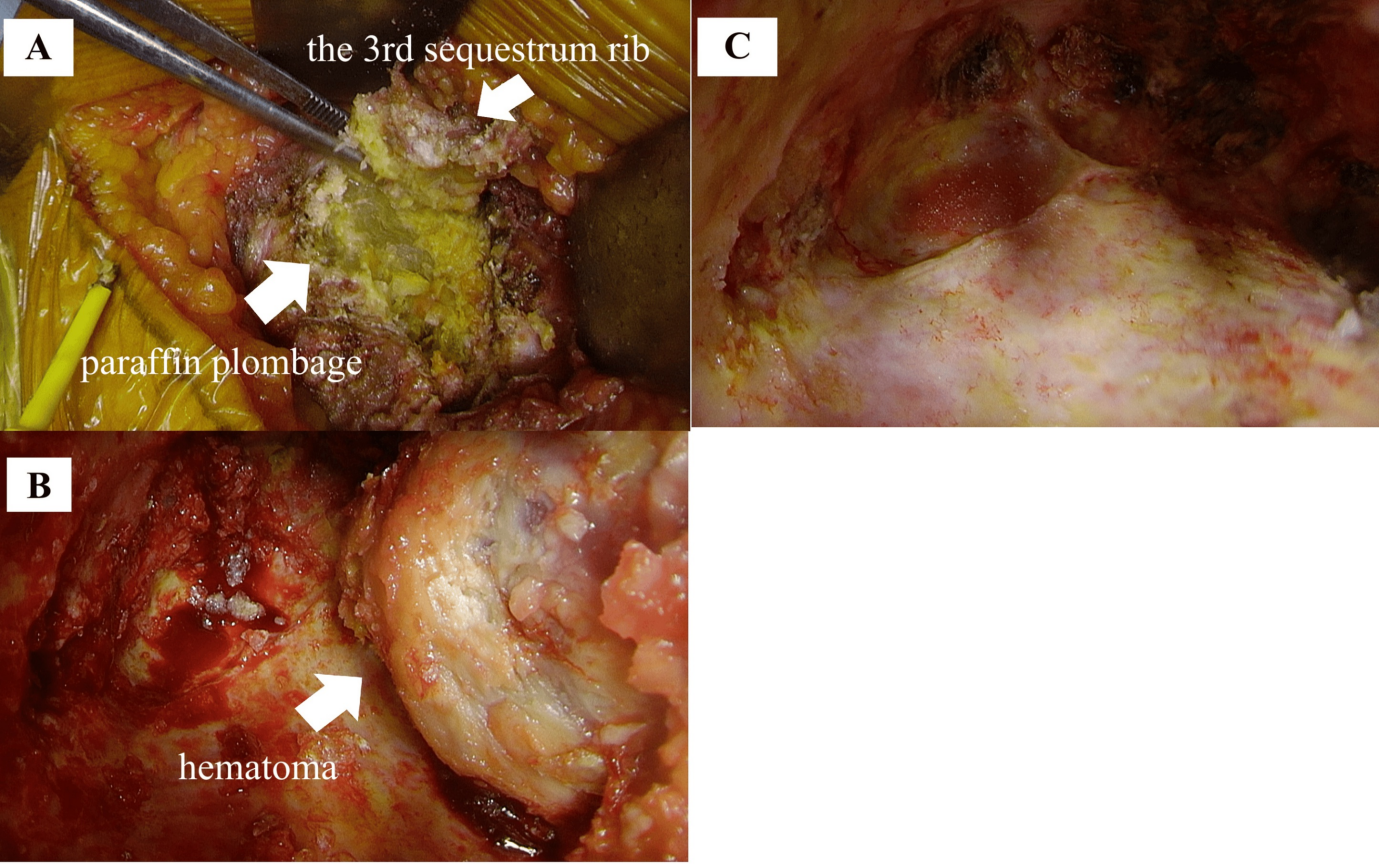
Operative findings (A) Paraffin was removed from the plombage space. (B) Solid hemorrhagic mass is on the mediastinal side in the space. (C) Removed hematomas from the space.

The surgical approach was through the third intercostal space; however, the third rib was sequestrated, and the paraffin was exposed (Figure 3A). We resected the perineurium and accessed the plombage space. The yellow paraffin was filled in solid form and removed. A hemorrhagic mass surrounded by a smooth, thickened capsule was identified on the mediastinal side (Figure 3B). When the mass with the capsule was removed, it bled from inside the mass easily. The plombage space was irrigated with 15 L of saline solution until no floating paraffin remained (Figure 3C). The operative time was 153 minutes, and the blood loss was 100 mL.

Gross examination revealed old hematomas surrounded by a fibrous capsule.

**Figure 4:**
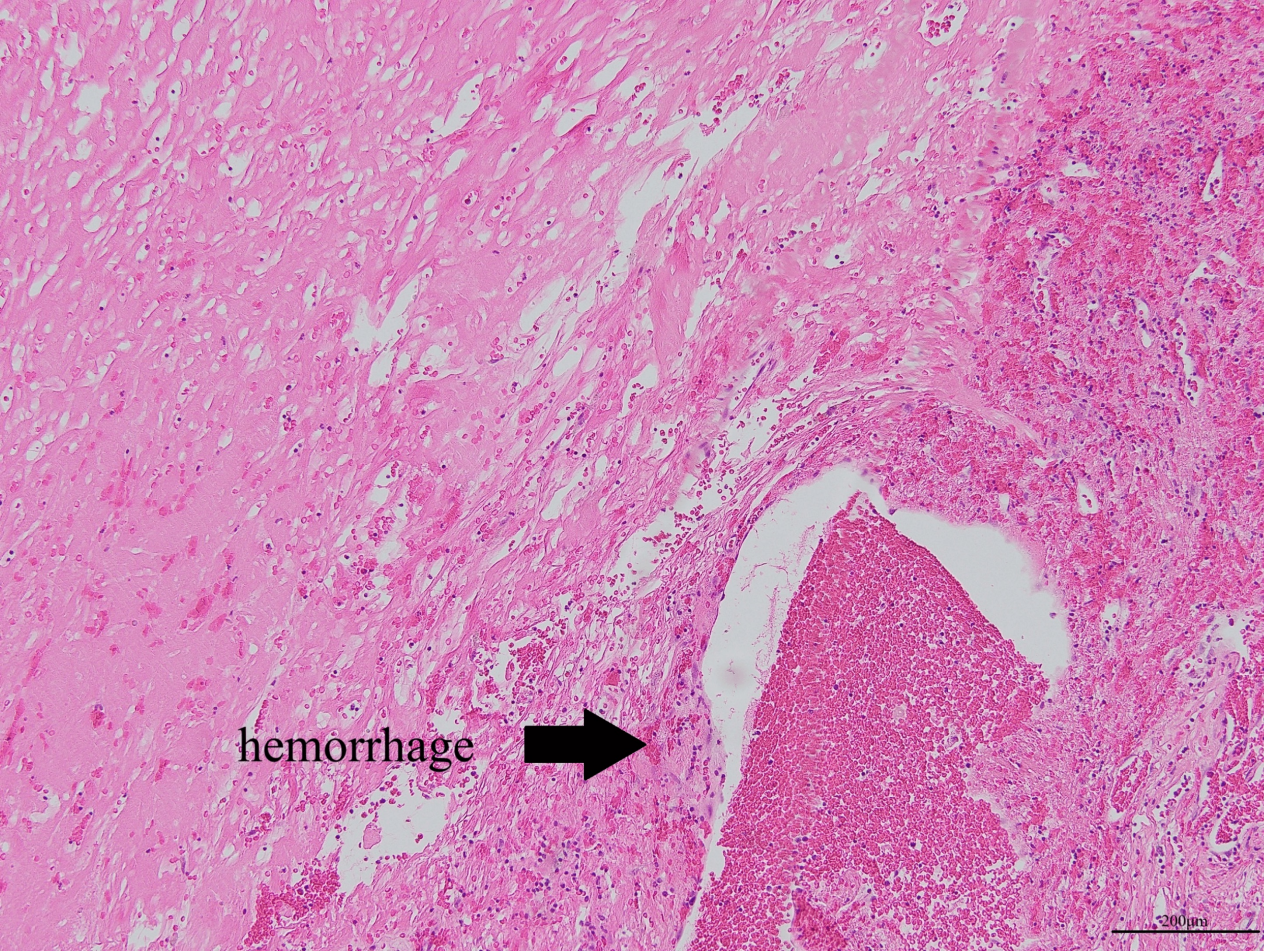
Pathological findings The pathological findings showed necrotic tissue with some hemorrhage (H-E stain, ×100)

Histopathological examination confirmed CEH with necrotic tissue and hemorrhage (Figure 4). Bacterial culture tests submitted at the time of surgery were negative for both the empyema wall and paraffin.

**Figure 5:**
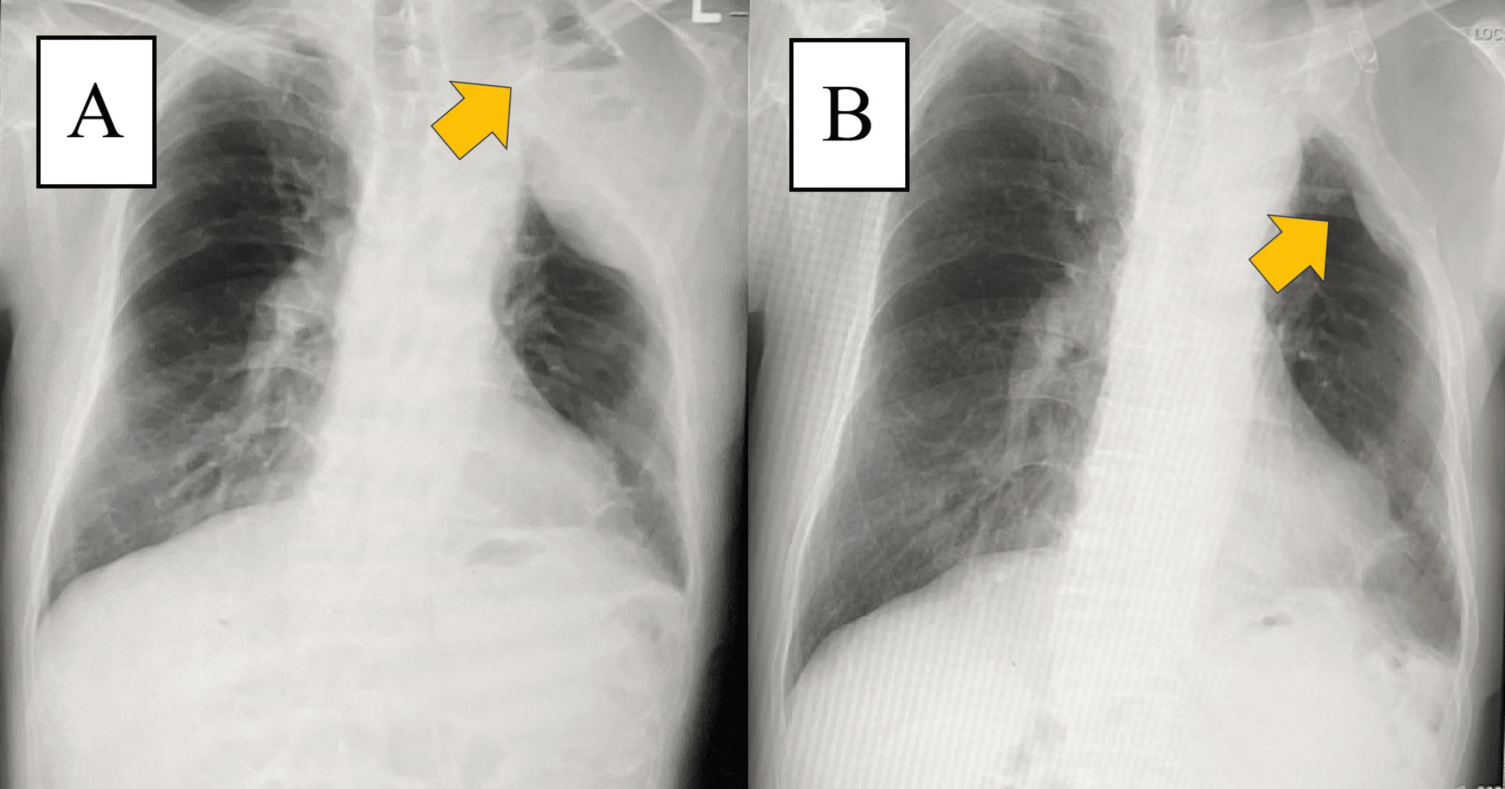
Postoperative chest X-ray findings (A) On the chest X-ray one month after surgery, a pleural effusion and an air-fluid level were observed in the left upper lung field (arrow). (B) On the chest X-ray two years after surgery, the pleural effusion decreased (arrow).

The patient was discharged from the hospital on postoperative day 8 without complications. Gradually, the chest pain improved, and it completely disappeared six months after the surgery. He had no recurrences during the two years of follow-up. On the chest X-ray one month after surgery, a pleural effusion and an air-fluid level were observed in the left upper lung field (Figure 5A). On the chest X-ray two years after surgery, the pleural effusion further decreased (Figure 5B).

## Discussion

Extraperiosteal paraffin plombage was developed as a collapse therapy for pulmonary tuberculosis, involving the placement of paraffin between the rib and periosteum. This treatment was considered safe with no serious early complications, and it produced an adequate and permanent collapse, thus resulting in a cure for pulmonary tuberculosis [7]. However, various late complications have been reported following paraffin plombage (Table 1) [2-6]. The onset of late complications varies between 20 and 50 years postoperatively. Cases of paraffinoma with spinal paralysis and IgG4-related disease have been reported after paraffin plombage [3,4]. CEH-associated pyothorax and pyothorax-associated lymphoma have also been reported [8,9]. In our case, while the appearance period of the mass was unknown, the patient became aware of left lateral chest pain 50 years after paraffin plombage. This is the latest-onset case and demonstrates that late complications could occur even after more than 50 years.

**Table 1:** Summary of the reported late complications of paraffin plombage

Author	Age (years)	Sex	Complications	Interval from plombage (years)	Treatment
Arai et al. [3]	79	M	Paraffinoma	47	Surgery
Tanaka et al. [2]	57	M	CEH*	22.5	Surgery
	44	M	CEH*	21	Surgery
	43	M	CEH*	21	Surgery
	65	M	CEH*	21	Surgery
	53	M	CEH*	17	Surgery
	50	F	CEH*	30.5	Surgery
	60	M	CEH*	27.5	Surgery
	67	M	CEH*	14	Surgery
Fujiwara-Kuroda et al. [6]	70s	M	CEH*	48	Surgery**
Horowitz et al. [5]	77	F	Migration and pyothorax	35	Surgery
Isoda et al. [4]	75	M	IgG4-related disease	54	Oral prednisolone
Our case	75	M	CEH*	50	Surgery
*chronic expanding hematoma **surgery and arterial embolization					

CEH was first described by Reid et al. in 1980 [10]. Repeated microvascularization and hemorrhaging caused by chronic aseptic inflammation can lead to hematoma and expansion of the space with organized tissues over decades. If left untreated, CEH can cause respiratory failure due to mediastinal compression, and in some cases, it is difficult to distinguish malignant diseases such as malignant lymphoma. Therefore, surgical treatment is required [7,11]. The optimal treatment for CEH is complete surgical removal of both the hematoma and its capsule, because incomplete removal of the hematoma may result in recurrence of the hematoma. In this case, CEH was suspected based on CT findings showing an enlarged plombage space and mass. The elevated NSE levels prompted consideration of malignant lymphoma in the differential diagnosis. However, pathological examination showed no evidence of malignancy, suggesting that chronic inflammation caused by paraffin led to CEH and plombage space enlargement.

This patient was suspected of malignancy and underwent surgery; the result was benign CEH, but the chest pain disappeared postoperatively. CEH is also a disease that can cause serious symptoms if enlarged, and surgical curettage should be performed if there are symptoms or mass formation after paraffin plombage.

## Conclusions

We report a case of CEH occurring 50 years after extraperiosteal paraffin plombage, which was successfully treated by surgical resection. This case highlights that CEH can develop long after extraperiosteal paraffin plombage, particularly when patients present with symptoms or mass enlargement in the plombage cavity. In such cases, differential diagnoses should include malignant tumors, such as malignant lymphoma. Surgical treatment is recommended not only to rule out malignancy but also to eliminate chronic inflammation.
